# Genetics and Molecular Basis of Congenital Heart Defects in Down Syndrome: Role of Extracellular Matrix Regulation

**DOI:** 10.3390/ijms24032918

**Published:** 2023-02-02

**Authors:** Nunzia Mollo, Roberta Scognamiglio, Anna Conti, Simona Paladino, Lucio Nitsch, Antonella Izzo

**Affiliations:** 1 Department of Molecular Medicine and Medical Biotechnology, University of Naples Federico II, 80131 Naples, Italy; 2 Institute of Experimental Endocrinology and Oncology, National Research Council, 80131 Naples, Italy

**Keywords:** Down syndrome, chromosome 21, congenital heart defects, extracellular matrix, *RUNX1*

## Abstract

Down syndrome (DS), a complex disorder that is caused by the trisomy of chromosome 21 (Hsa21), is a major cause of congenital heart defects (CHD). Interestingly, only about 50% of individuals with Hsa21 trisomy manifest CHD. Here we review the genetic basis of CHD in DS, focusing on genes that regulate extracellular matrix (ECM) organization. The overexpression of Hsa21 genes likely underlies the molecular mechanisms that contribute to CHD, even though the genes responsible for CHD could only be located in a critical region of Hsa21. A role in causing CHD has been attributed not only to protein-coding Hsa21 genes, but also to genes on other chromosomes, as well as miRNAs and lncRNAs. It is likely that the contribution of more than one gene is required, and that the overexpression of Hsa21 genes acts in combination with other genetic events, such as specific mutations or polymorphisms, amplifying their effect. Moreover, a key function in determining alterations in cardiac morphogenesis might be played by ECM. A large number of genes encoding ECM proteins are overexpressed in trisomic human fetal hearts, and many of them appear to be under the control of a Hsa21 gene, the RUNX1 transcription factor.

## 1. Introduction

Congenital heart defects (CHDs) refer to a range of heart developmental anomalies occurring in ≈1% of live births [1,2]. CHDs are present with approximately a 50% recurrence in Down syndrome (DS) subjects [3,4]. The most frequent anomalies in DS are endocardial cushion defects [5] such as atrioventricular canal defects (AVCD), ventricular septal defects (VSD), atrial septal defects (ASD), patent ductus arteriosus (PDA) and tetralogy of Fallot (ToF) [6,7]. Since CHD is a very frequent sign in DS, it seems plausible that an altered expression of gene(s) mapping to chromosome 21 (Hsa21) might play a role in cardiac development.

**Figure 1:**
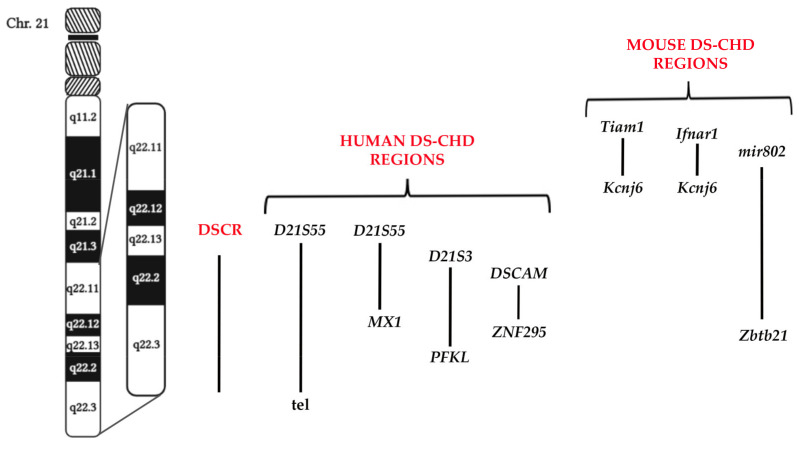
Schematic representation of DS phenotype critical regions. The ‘Down Syndrome Critical Region’ (DSCR) [8,9,10] and human [11,12,13,14,15] and mouse [19,20,21] DS–CHD regions with corresponding boundaries have been defined using the GRCh38/hg38 UCSC assembly.

Some years ago, independent studies suggested that most signs of the DS phenotype were associated with three copies of chromosome band 21q22.2–22.3, which was named the Down syndrome critical region (DSCR) [8,9,10]. Later, a critical region responsible for the cardiac phenotype in DS patients, the DS-CHD, was established in a 9 Mb Hsa21 region including *D21S55* through the telomere [11]. This region was then narrowed down to approximately a 4 Mb region, from *D21S55* to *MX1* [12,13]. In 2001, Barlow et al. proposed a new candidate DS-CHD region spanning from *D21S3* to *PFKL* [14]. This segment was further limited to a region spreading less than 2 Mb from *DSCAM* to *ZNF295* [15] (Figure 1). Recently, a more restricted region spanning about 1 Mb and containing three protein-coding genes (*DSCAM*, *BACE1* and *PLAC4*), was proposed [16].

Most of these studies, based on human partial trisomies, are hampered by the fact that the karyotype of the examined individuals was complex, including other chromosome anomalies, and the phenotype was quite heterogeneous and rare. For this reason, mouse strains have been generated to recapitulate the pathology and genetics of DS-CHD [17,18]. In the DS mouse model Dp(16), Liu et al. identified a genomic region associated with CHD similar to that observed in DS subjects [19]. This region, which spans from *Tiam1* to *Kcnj6* and includes 52 Hsa21 ortholog genes, was further narrowed to 3.7 Mb from *Ifnar1* to *Kcnj6* (35 Hsa21 ortholog genes) [20]. Lana-Elola et al., using a mapping panel of seven mouse strains, proposed as DS-CHD a genomic region from *Mir802* to *Zbtb21*, partially overlapping what was previously described by Liu [21] (Figure 1).

In any case, whatever the critical region for CHD in DS, multiple findings suggest that cardiac defects in DS are not the result of the overexpression of a single gene, but of a set of them [22].

In this review, we address the role of genes mapping to Hsa21 and other chromosomes in the development of the heart and in molecular mechanisms responsible for CHD. Since the extracellular matrix (ECM) is known to play a crucial role in the morphogenesis of the heart, we focus on those genes that code for proteins involved in ECM assembly and function, and whose expression is dysregulated as a consequence of Hsa21 trisomy.

## 2. Candidate Genes for CHD in DS

### 2.1. Genes Mapping to Hsa21

As trisomy 21 (T21) is the genetic abnormality most commonly associated with heart defects, studying the contribution of Hsa21 genes in the development of the heart could be an entry point for better understanding the pathogenesis of CHD.

Many experiments have been carried out to understand the molecular mechanisms leading to CHD in DS. Several genes mapping to Hsa21 are involved in embryonic cardiogenesis. Moreover, it has been proposed that altered Hsa21 gene dosage can magnify the effects of either pathogenic mutations or polymorphisms in other genes [6]. The transcriptional profile of human hearts from DS fetuses demonstrated that most Hsa21 genes are overexpressed in the developing heart due to gene dosage [23].

Some evidence suggests the existence of genes on Hsa21, involved in cell adhesion, which likely play an important role in valvulo-septal morphogenesis. If overexpressed, these genes may cause cardiac defects [14,24,25]. One of these genes is *DSCAM*, a member of the immunoglobulin superfamily of cell adhesion molecules (Ig-CAMs) [26]. It is expressed during cardiac development before endocardial cushion fusion. Its overexpression, due to the trisomy, is responsible for increased cell–cell adhesion [14,27]. This may induce alterations in the cushion development [14].

In a similar way, the overexpression of collagen VI, two of three chains of which map to Hsa21, has been associated to cardiac septal defects in DS [28,29]. Fetal human heart staining demonstrated that collagen VI is present in the atrioventricular (AV) cushions and that its expression is higher in T21, possibly altering their development [29]. Collagen VI is an activator of discoidin domain receptors (DDRs) [30], which regulate cell–collagen interactions and, in turn, induce the expression of metalloproteases and other ECM proteins [31]. DDR2 expression has been detected by confocal microscopy in the developing heart, within the cardiac cushions and eventually within the septum [32]. *DDR1* and *DDR2* are upregulated in trisomic heart samples [23].

Interestingly, septal defects have been observed when both *DSCAM* and *COL6A1* are co-expressed in the murine heart, indicating that the overexpression of these two genes may exert synergistic effects on cardiac defects [33], even though Kosaki et al. argued that triplication of the *DSCAM* gene alone might be the cause of cardiac defects in DS [34].

Two other Hsa21 genes are potential contributors to AVSD in DS patients: the dual-specificity tyrosine phosphorylation-regulated kinase 1A (*DYRK1A*), and the regulator of calcineurin 1 (*RCAN1*), also known as *DSCR1*. Synergistically, both cause a decrease in the activity and levels of the transcription factors belonging to the nuclear factor of active T cell (NFATc) family [35].

*DYRK1A* encodes a nuclear serine/threonine kinase that primes substrates for phosphorylation by GSK3, which phosphorylates NFATc proteins in the nucleus, resulting in their inactivation and export [36].

*RCAN1* encodes a binding protein that negatively controls calcineurin activity [37]. Calcineurin is a unique serine/threonine protein phosphatase under the control of Ca^2+^/calmodulin: an increase in intracellular Ca^2+^ leads to the activation of calcineurin [38], which then dephosphorylates NFATc proteins, causing their nuclear entry and assembly with partner proteins (NFATn) to form NFAT transcription complexes [39]. The NFAT signaling pathway, a critical regulator of vertebrate development and organogenesis [40], is important for the cardiac valve and septum morphogenesis [41,42,43]. NFATc mutant mice manifest vascular and cardiac morphogenic defects [36]. Striking similarities have been detected between the phenotypic features of DS and mice carrying deletions of genes encoding components of the NFAT signaling pathway, and 65% of NFATc1–4-null mice have endocardial cushion defects [35].

Acting as a negative regulator of the NFAT signaling pathway, the overexpression of *DYRK1A* and *RCAN1* genes in DS may be considered a putative cause of cardiac development defects in DS patients [44,45]. *NFATc* genes were found to be downregulated in the heart tissues of fetuses with DS, while *DYRK1A* was upregulated [23]. *RCAN1* expression was greatly variable and not overall significantly dysregulated in DS fetal hearts, but its expression was inversely correlated with that of *NFATc3* [23].

Interestingly, DYRK1A and RCAN1 are also crucial regulators of Synaptojanin 1 (*SYNJ1*), another Hsa21 gene encoding a polyphosphoinositide phosphatase. The former, phosphorylating SYNJ1, the latter implicated in its dephosphorylation, finely modulate the SYNJ1 activity [46,47].

SYNJ1 is a key player of early endosomal compartments, regulating their homeostasis and functions in different cell types [48,49,50]; on the other hand, a dangerous liaison between Synj1 and endosomal trafficking has been observed in DS [51,52]. Compelling evidence has shown that endosomal transport is critical for heart functions [53], controlling the localization and turnover of cardiac proteins, such as cardiac pacemaker channels [54] and sodium channels [55], suggesting a potential role of this pathway in the cardiac defects of DS.

The notion that only half of DS subjects present CHD suggests that some other events, such as copy number variations (CNV), SNPs, or other genetic anomalies, may concur with trisomy to alter heart development [56].

CNVs of Hsa21 tracts have been associated with AVSD risk in DS. The analysis of CNV in DS subjects with AVSD revealed three regions associated with an AVSD risk. Two of them are located within the previously identified DS-CHD region on Hsa21: the former maps near the *RIPK4* gene, and the latter in the *ZBTB21* (previously *ZNF295*) gene, suggesting the potential role of these genes in the pathogenesis of CHD in DS [56]. This last gene was overexpressed in the hearts of fetuses with CHD when compared with DS hearts without CHD (dataset from ref. [21], unpublished results). The *ZBTB21* gene has also been shown to interact with *PPP2R2B*, which regulates the WNT/β-catenin signaling pathway in drosophila. This pathway is required for cardiac differentiation in human embryonic stem cells [57].

Finally, relationships have been established between SNPs in two interferon receptor genes (*IFNAR1* and *IL10RB*) and CHD in subjects with DS [58]. These receptors map to the 3.7 Mb critical genomic region associated with DS heart defects described by Liu et al. [20].

### 2.2. Genes Mapping to Other Chromosomes

The role of non Hsa21 genes in causing CHD has been demonstrated in humans and experimental mice by studies not related to DS.

Rare variants in *BMP4*, *CRELD1*, *CRELD2*, *FBLN2*, *FRZB, GATA4* and *GATA5* genes have been found in individuals with DS and complete AVSD, suggesting that rare genetic variants in these genes, incompletely penetrant on an euploid background, may act synergistically with T21 to increase the risk for AVSD in DS [59,60,61,62]. *CRELD1* (mapping to 3p25) encodes a cell surface protein that might participate in cell adhesion. Maslen et al. found two *CRELD1* missense mutations in children with DS and AVSD, implying its role in the pathogenesis of the disease [5]. A pathway analysis proved that all these genes, together with the DDRs cited above, are somehow associated with the vascular endothelial growth factor-A (VEGF-A) pathway [60,63,64,65,66].

Several studies highlight the potential contribution of the VEGF-A and calcineurin/NFAT pathways to CHD development in DS patients [37,60]. VEGF-A is a strong mitogen known as a regulator of AV valvuloseptal morphogenesis, wherein it is critical for the formation of the AV endocardial cushions and helps the morphogenesis of those primordial structures into the AV valves [67,68]. Studies in animal models have shown that altered expression levels of VEGF-A during heart development are associated with CHD, including AVSD [69,70,71,72], and that *VEGF-A* overexpression in mouse embryos triggers severe heart developmental abnormalities [73].

An imbalance of overexpressed Hsa21 genes is known to affect the expression of genes located in different chromosomes [23,74,75]. More than 400 genes that do not map to Hsa21 were found to be dysregulated in the heart tissues of fetuses with DS [23]. It was observed that the most dysregulated category consisted of genes coding for mitochondrial proteins belonging to all five complexes, as well as proteins involved in mitochondrial biogenesis and function. The fact that these nuclear-encoded mitochondrial genes (NEMGs) were all downregulated suggested that the corresponding proteins and enzymatic activities might be reduced in DS cells, and that mitochondrial function could be consequently impaired [76]. It was indeed determined that the mitochondria of DS fibroblasts presented an altered ultrastructure, with breaks in the inner membrane and alterations in the mitochondrial cristae, a reduced oxygen consumption rate (OCR) and mitochondrial membrane potential, and an increase in intracellular ROS production [76]. These findings are relevant in light of the fact that alterations of mitochondrial morphology have been shown to affect cardiomyocyte differentiation, apoptosis and autophagy [77], and that mitochondrial activity and energy conversion play a central role in the normal function of the heart. Mitochondrial dysfunction is considered to be one of the relevant mechanisms that plays a role in the pathogenesis of cardiovascular diseases [22,78]. Furthermore, the comparison between fibroblasts from DS fetuses with and without heart defects led to the intriguing observation that mitochondrial dysfunction was more severe in fibroblasts from cardiopathic trisomic fetuses, which presented a more pronounced pro-oxidative state [76], suggesting that mitochondria may be an interesting target in the pathogenesis of CHD. It is worth mentioning that the NEMGs downregulated in T21 fetal hearts were under the control of *PGC-1α*, which is, in turn, regulated by the Hsa21 gene *NRIP1*. The attenuation of *NRIP1* expression by siRNAs in trisomic fibroblast [79] or the promotion of *PGC-1α* activity via metformin [80] or pioglitazone [81] were able to restore the level of expression of NEMGs and to counteract mitochondrial dysfunction [82,83]. It will be of interest to further explore the role of *NRIP1*, *PGC-1α*, NEMGs and mitochondrial dysfunction in the pathogenesis of CHD.

Two of the genes that were found to be downregulated in the heart tissues of fetuses with T21 [23], namely *TBX20* and *SRF*, deserve particular attention.

The cardiac T-box factor *Tbx20* is expressed in the early cardiac progenitor region, endocardium and myocardium, endothelial cells of the outflow tract (OFT), endocardial cushions and AV cushions, the precursor structure of cardiac valves and the atrioventricular septum. It directly interacts with Nkx2-5, GATA4 and GATA5 in regulating gene expression in the developing heart [84] and acts as a key upstream regulator for a variety of other genes, such as *Bmp2*, *Tbx3* and *Hand1*, in the early atrio-ventricular channel (AVC) development [85]. *Tbx20* is, therefore, essential for AVC patterning and cushion formation, playing crucial roles in these two important aspects to coordinate early heart development [85]. Mutations in *TBX20* are widely associated with the complex spectrum of CHD in humans, which includes defects in chamber septation, chamber growth and valvulogenesis [84].

Serum response factor (SRF) is a major transcription factor that controls both embryonic and adult cardiac development. *SRF* expression is needed throughout development, from the first mesodermal cell in a developing embryo to the last cell damaged by infarction in the myocardium. *Srf* indirectly regulates *Hand2* expression through a miRNA-mediated mechanism [86]. The precise regulation of *SRF* expression is critical for mesoderm formation and cardiac crescent formation in the embryo, and altered *SRF* levels lead to cardiomyopathies in the adult heart, suggesting its vital role in cardiac development and disease [87].

### 2.3. Role of ncRNAs in the Pathophysiology of CHD in DS

Several microRNAs mapping to Hsa21 are overexpressed in DS [88,89]. *MiR-99a*, *let-7c*, *miR-125b-2*, *miR-155* and *miR-802* were found to be overexpressed in the cardiac tissue of patients with T21 [90,91]. It has been demonstrated that cardiomyogenesis is controlled by the *miR-99a/let-7c* cluster [88]. When overexpressed, *let-7c* promotes cardiomyogenesis by upregulating the genes involved in mesoderm specification (*T/Bra* and *Nodal*) and cardiac differentiation (*Mesp1*, *Nkx2.5* and *Tbx5*), repressing its direct target *EZH2*. On the contrary, *miR-99a* represses cardiac differentiation, targeting *Smarca5* in mouse. Indeed, *EZH2* and *SMARCA5* were found to be downregulated in DS fetal hearts, suggesting that they might participate in CHD pathogenesis [88].

Another let-7c target, *SLC25A4/ANT1*, was identified and validated by Izzo et al. [91]. This gene, which is downregulated in DS fetal hearts [23], is the main translocator of ADP/ATP across the mitochondrial membrane; therefore, its repression might have a potential negative impact on mitochondrial function. It is known that mitochondrial dysfunction might affect several DS features, including cardiac alterations [22,92].

It was reported that Hsa21 miR-155-5p affects mitochondrial biogenesis by targeting the mitochondrial transcription factor A (*TFAM*), a gene that was found to be downregulated in trisomic hearts [93]. TFAM is a nuclear-encoded protein that controls the transcription and maintenance of mtDNA and, therefore, mitochondrial biogenesis.

Among miRNAs that do not map to Hsa21, *miR-1* has been shown to regulate cardiac differentiation and control heart development in mice determining miRNA-guided translational inhibition and repression of the cardiac transcription factor Hand2 [94]. Particularly, *miR-1* is regulated by the transcription factor SRF [95], which is downregulated in DS fetal hearts [23]. Its expression is consequently downregulated, causing an excessive synthesis of *HAND2* (unpublished data), which is a possible cause of VSD.

Recent investigations have focused on the role of lncRNAs in the pathophysiology of CHD in DS. For instance, a genome-wide association study in DS subjects identifies in the FLJ33360 lncRNA a common SNP variant associated with an increased risk of DS-associated AVSD. FLJ33360 maps to chromosome 7. Interestingly, its adjacent gene *MED10* has been associated with cardiac defects [96] and participates in heart valve formation in zebrafish by affecting the expression of the transcriptional regulator Tbx2b in the AV myocardium [97].

## 3. Role of ECM Regulation in the Development of CHD in DS

The early stages of development of the cardiovascular system, as for other body systems, are characterized by a primordial ECM, whereas later developmental stages are characterized by resorption of this matrix and transition to a mature collagen- or elastin-rich ECM, a process that continues into the neonatal period [98]. The cardiac ECM is a dynamic, robust, and functionally versatile component that forms the non-cellular part of the cardiac muscle. It acts not only as an architectural scaffold that supports the cardiac cells, but also actively participates in the development and differentiation of cardiac and vascular cells [99]. Indeed, the cellular response to changes in ECM can trigger epithelial-mesenchymal transformation (EMT), the developmental process thought to be responsible for heart valve and septa development [100].

Endocardial cushions consist of an ECM jelly, containing highly proliferative valve progenitor cells and susceptible to the continual remodeling of its components, which is necessary for subsequent morphogenetic events [101]. The presence of ECM jelly is fundamental in the ventricular wall and endocardial cushions during their formation.

The transcriptional profile of human hearts from DS fetuses demonstrated that genes coding for ECM proteins, proteoglycans, collagens and multi-adhesive matrix proteins are over-represented among the upregulated ones [23]. A meta-analysis of 45 heterogeneous DS data sets [102] confirmed this trend, as a functional analysis of the 324 genes consistently upregulated in trisomic samples indicated that 37 of them belonged to the ECM Cell Component GO category. A significant dysregulation of genes coding for ECM components has also been reported in other meta-analysis studies [103,104].

Given this fundamental role of ECM in heart development, it is conceivable that the consequence of gene dysregulation on ECM composition and assembly may contribute to congenital anomalies, including cardiac defects [4,29].

More than 40 genes encoding ECM proteins were found to be upregulated in DS fetal heart tissues [23]. This group included genes mapping to Hsa21, such as members of the ADAMTS protein family (*ADAMTS1* and *ADAMTS5*), *APP*, *JAM2* and collagens (*COL6A1*, *COL6A2* and *COL18A1*), which are dose-dependently upregulated in trisomic samples. It also includes genes that do not map to Hsa21, such as collagen type I (*COL1A1* and *COL1A2*), type III (*COL3A1*), type V (*COL5A1* and *COL5A2*), type 9 (*COL9A2* and *COL9A3*), type 13 (*COL13A1*), type 14 (*COL14A1*), type XV (*COL15A1*), versican (*VCAN*), fibronectin (*FN1*), fibulin (*FBLN1*), metalloproteases (*MMP2* and *MMP11*) and several adhesion molecule genes, which are likely regulated by the overexpression of Hsa21 genes. All these genes are supposed to affect cell adhesion properties, possibly determining an increase in adhesiveness [23]. Their function and potential role in CHD are listed in Table 1.

Aberrant cell adhesion, migration and proliferation have been demonstrated using a trans-chromosomic mouse model, which contains a supernumerary human chromosome 21 [141]. Possibly, increased adhesion and aberrant migration in DS cells are independent of each other; hence, they might be caused by different mechanisms and different Hsa21 genes, but both events might be required for the AVC defect to develop [141].

The increased adhesiveness of T21 cells has for many years been considered as a main cause for the failure of the embryonal endocardial cushion to septum fusion, which provokes the persistence of the AVC and/or the perimembranous VSD [142]. To better understand the molecular mechanisms, the integrin-mediated cell adhesive properties on FN, COLI and COLVI of skin fibroblasts isolated from DS and non-DS individuals have been compared [143]. All DS fibroblasts displayed an aberrantly increased adhesive capacity for COLVI if compared to non-DS fibroblasts with a mechanism dependent on the altered activation state of the β1 integrin [143].

Collagen VI is composed of three alpha chains, two of which are encoded by Hsa21 genes. It is a component of ECM responsible for anchoring cells within the three-dimensional tissue space by binding to cell surface integrins and other structural matrix components [144]. Its expression has been documented in the developing AV cushions and the adult AV valves of several species [29,112,113,114].

Collagen XVIII is highly expressed throughout the connective tissue core of the endocardial cushions and in forming the AV valve leaflets. It was closely associated with the EMT of endothelial cells into mesenchymal cushion tissue cells and was localized around these cells as they migrated into the cardiac jelly to form the initial connective tissue elements of the valve leaflets [116].

Other collagen proteins are expressed in the developing heart [109]. Collagen type I is normally expressed in AV valves and in the aortic wall and it is important for this vessel’s elasticity and integrity [109]. Collagen type III is also expressed in the vasculature of mice. The *Col3a1* knockout mouse dies late in its adult life from the rupturing of blood vessels [128]. Recently, *COL3A1* was defined as the most common causative gene in a cohort of 121 CHD patients [129]. *Col5a1* knockout mice are embryonically lethal for cardiovascular insufficiency [130]. Finally, collagen type XV plays a role in matrix remodeling in the heart and participates in the organization of the collagen fibrils [133]. *Col15a1* knockout mice also revealed that it is required for proper circulation in specific microvascular beds. For these reasons, it should be considered as a candidate gene for involvement in human familial cardiomyopathies [133].

The Hsa21 gene *JAM2* encodes a cell membrane protein with immunoglobulin-like domains that is concentrated at cell-to-cell junctions in the heart endothelial cells of both large and small vessels, and it has been implicated in angiogenesis defects in Tc1 mice [117]. Jam2 plays a necessary role in the cross-talk between trisomy and Creld1 in Ts65Dn. Indeed, when present at 0, 1, 2, or 3 copies, it has no effect on heart development. However, when *Jam2* is trisomic and overexpressed, there is an increased penetrance of septal defects in mice with only one copy of *Creld1* [118].

VCAN may influence cell adhesion, proliferation, migration and survival by binding to several other ECM components. Its role is necessary for cardiac cushion formation, atrioventricular valve development, ventricular septation and OFT development [140]. VCAN is cleaved by MMPs and members of the ADAMTS family [106]. This process is necessary for AV cushion, OFT and trabecular development [106,107]. The dysregulation of ADAMTS proteins and MMPs is supposed to compromise this process.

FN1, a multi-domain ECM protein that interacts with multiple integrins, proteoglycans, collagens and fibrins [145], is expressed early in embryonic development [137,146]. As cardiac development progresses, it is expressed in the dorsal aortae, pharyngeal arch arteries, endocardium [137,139] and in the mesenchyme of the endocardial cushions, where it is required for EMT-mediated development [136,138].

FBLN1 can bind a variety of ECM molecules, including aggrecan and versican, which are known targets for cleavage by ECM metalloproteases [106,135,147]. At E9.0 to E9.5 in the mouse, fibulin-1 is expressed in the dorsal neural tube and is associated with the developing cardiac neural crest cells. By E10.5, fibulin-1 is found in pharyngeal arches 3 and 4 and the distal OFT cushions [134,135]. *FBLN1* is also expressed throughout the ECM of the AV cushions (ED9.5-ED14.5) [106]. It has been implicated in the regulation of cell migration of the AV mesenchyme [105], possibly due to its role as a cofactor to ADAMTS1, in the cleavage of ECM molecules known to promote migration [105,106].

## 4. The Hsa21 Transcription Factor RUNX1 Regulates the Expression of ECM Components

Searching for Hsa21 genes that may contribute to ECM gene regulation, RUNX1 (runt-related transcription factor 1) has been identified as a possible candidate according to the following evidence [148]: (i). the analysis of experiments in which *RUNX1* gene expression was modulated has shown that ECM is one of the most affected categories [149]; (ii). *RUNX1* and several ECM genes, located or not on Hsa21, are upregulated in human DS fetal hearts and fibroblasts [23], and most of them have consensus sequences for *RUNX1* in their promoters; (iii). the attenuation of RUNX1 by siRNAs decreased the expression of 11 out of 14 ECM genes that are overexpressed in DS fetal fibroblasts (*ADAMTS5*, *APP*, *COL6A1*, *COL6A2*, *COL18A1*, *COL5A1*, *ECM2*, *FN1*, *FBLN1*, *MMP2* and *VCAN*) and increased the migratory capacity of trisomic fibroblasts, which are characterized by a migration defect compared to euploid controls [148]; (iv). the *RUNX1* gene is included in the 3.7 Mb minimal critical region for DS-CHD, as described by Liu et al. [20].

The *RUNX* genes encode the α-subunits of a family of transcription factors that orchestrate proliferation, differentiation and cell survival in multiple lineages. In mammalian species, three α-subunits exist, known as RUNX1, RUNX2 and RUNX3, each with its own distinct spatial-temporal and tissue-specific pattern of expression [119,150]. Although each RUNX protein interacts with the same target consensus sequence, they display distinct and non-redundant biological functions [150]. In the developing embryo, RUNX1 is the most broadly expressed of all the RUNX proteins and is expressed in a range of tissues, including the mesenchymal tissue of the heart and in vascular tissue [119].

Several studies have ascribed to RUNX1 an important role in regulating ECM genes, cell adhesion and migration [151,152,153,154].

In the murine hemogenic endothelium, more than 100 genes were bound by RUNX1 and positively correlated with its expression. They were clearly associated with cell adhesion, such as α and β integrins, cellular movement and interaction with ECM, such as Adamts family genes and collagens. Overall, integrin signaling was the top enriched canonical pathway influenced by *Runx1* modulation [151]. Several genes encoding cell surface or extracellular ligands, involved in cell-matrix adherence, have been defined as candidates for direct Runx1 regulation [153]. Furthermore, *RUNX1* knockdown in breast cancer cells resulted in the downregulation of genes belonging to ECM-related categories. This correlation was confirmed by the presence of RUNX1 binding sites in dysregulated gene promoters [154].

Finally, transcriptome and proteome profiling of trisomic neural cells revealed the dysregulation of several genes belonging to collagen, cell-adhesion, ECM–receptor interactions and integrin complex clusters. The most consistent upregulation during differentiation was identified for *RUNX1* [155].

Genes belonging to the RUNX family are transcriptional factors for collagen proteins [156,157,158], including COLIV. Collagen IV protein levels were found to be increased in trisomic fibroblasts, and decreased upon RUNX1 attenuation [148]. The overexpression of RUNX1 in hepatocellular carcinoma cells elevated COL4A1 expression, while its knockdown in SMMC7721 and SK-Hep1 cells significantly decreased its expression level [159]. Collagen IV, the main component of the basement membrane in the heart, plays a major role in the cell–matrix interaction, thus regulating the cell differentiation, migration, proliferation, adhesion and signaling cascade [160].

The expression of *RUNX1* positively correlated with multiple molecules of the MMP family in colorectal cancer [161] and other human tumors [162,163,164].

Silencing of *RUNX1* expression demonstrated a significant decrease in the expression levels of *MMP1*, *MMP2*, *MMP9*, *MMP19* and *VEGFA* [164].

The importance of RUNX1 in the development of the vasculature is highlighted in the phenotype observed in *Runx1* KO mice (Table 1). In the heart, these mice have an underdeveloped coronary plexus and smaller ventricular free wall vessels [124]. This coincides with changes in the heart structure, including ventricular septal defects and the development of a thin myocardium [124].

The expression of *RUNX1* in the neonatal heart is higher compared with adult heart tissue [122]. Although the reasons for this are unexplored, it is interesting to note that genes with RUNX1-binding sites within their promoter region are over-represented in the collection of genes that become methylated during the first week of life [165]. In this setting, increased gene methylation may be important in the maturation process by switching off genes necessary for heart development to support transition to a more adult phenotype [125,166].

## 5. Conclusions

**Figure 2:**
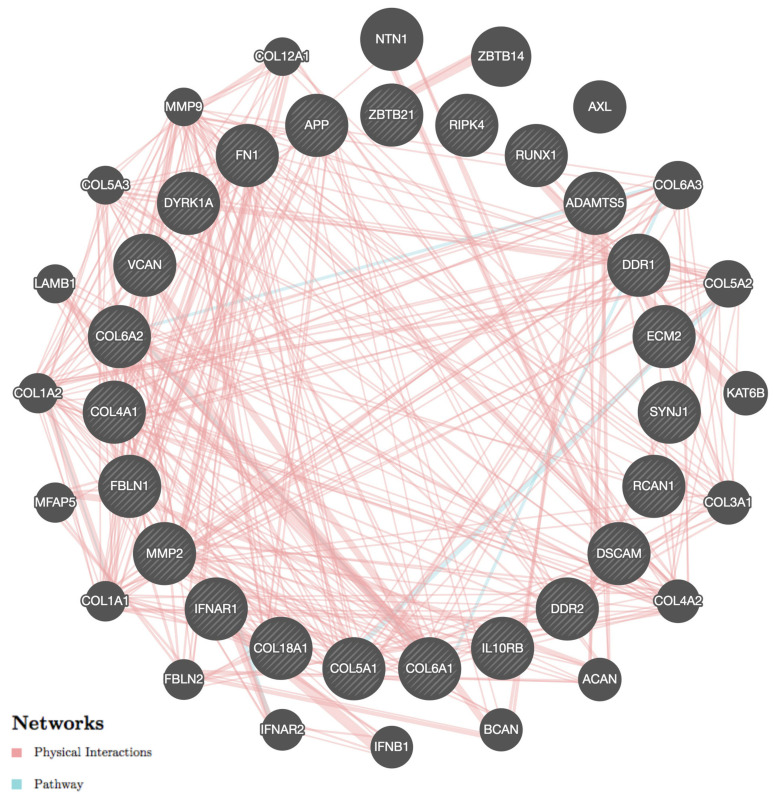
Interactions among genes involved in CHD. The figure illustrates the physical interactions and participation in the same pathway of genes involved in CHD generation, obtained using the GeneMANIA software (version 3.6.0) [174,175] (available at: https://genemania.org/ (accessed on 27 January 2023)). The genes discussed in this review are represented by the striped circles depicted in the inner ring. They are connected to each other by lines of different colors, each indicating a specific type of interaction: pink for physical interactions and light blue for pathways. The outer ring shows genes closely related to those in the inner ring (according to the GeneMANIA software). These genes are represented as circles of different sizes according to their ranking, which is achieved by scoring them using label propagation. By inputting the names of the genes in GeneMANIA, it is possible to generate an image featuring active links that provide additional information about all interactions and relevant literature references.

In this review we addressed the role of genes mapping to Hsa21 and other chromosomes in molecular mechanisms involved in CHD. The genes we discussed are linked by many different types of interactions (Figure 2), which may help to understand how they cooperate in the morphogenesis of the heart and the generation of its malformations.

The overexpression of Hsa21 genes is clearly responsible for the pathogenesis of CHD in DS, either directly or in an indirect manner, by dysregulating the expression of genes or miRNAs involved in embryonic cardiogenesis. A direct effect is exerted by the upregulation of Hsa21 genes, such as *DYRK1A* and *RCAN1*, which may affect septum morphogenesis by altering the NFAT signaling pathway; *DSCAM*, which increases cell adhesion; *COLVI*, which activates key ECM genes during cushion development; *NRIP1*, which controls mitochondrial function by regulating PGC-1α and the expression of NEMGs. The alteration of pathways resulting from the overexpression of these genes could be studied in an experimental model of T21 iPSCs [167], which can be differentiated into cardiomyocytes.

As an indirect effect, gene expression studies demonstrated in DS tissues and models the dysregulation of non-Hsa21 genes. The downregulation of genes belonging to the core network of cardiogenesis, such as *SRF*, *TBX20* and miR-1, and the global downregulation of genes encoding mitochondrial proteins—as well as the overexpression of genes encoding ECM proteins—have been documented in the heart of DS subjects [23].

The dysregulation and pathogenic mutations or polymorphisms of genes involved in cardiogenesis may concur to generate CHD. Rare variants of genes either belonging or associated to the VEGF-A pathway, as well as the CNV of Hsa21 tracts located within the previously identified DS-CHD region, have been found in DS subjects with AVSD, suggesting that they may increase the risk of CHD.

Quantitative and qualitative changes in the deposition of ECM molecules are crucial for tissue morphogenesis and homeostasis [168,169]. For this reason, the dysregulated expression and/or organization of ECM components in DS may be responsible for altered heart morphogenesis [29,170,171].

The overexpression of ECM-related genes in DS, postulated since 1998, when ultrastructural findings showed an extracellular precipitate containing glycosaminoglycans in the skin of DS human fetuses [172], was then demonstrated by gene expression profiling [23,102]. Specific changes in the expression and accumulation of ECM components have also been observed during human cardiomyocyte differentiation from trisomic embryonic stem cells in culture [140]. The ECM plays an important role in the development of the heart, in which the cardiac jelly, an acellular and ECM-rich space that separates the myocardial and endocardial cell layers in the primitive heart [173], is critical in heart septation and valvulogenesis [101]. It seems conceivable that even a small dysregulation of multiple ECM proteins involved in cardiogenesis may have profound effects on the proper formation of the atrioventricular septum and outflow tract, resulting in cardiac defects such as those observed in DS.

It has been proposed and demonstrated that the upregulation of *RUNX1*, a gene mapping to Hsa21, contributes to the overexpression of ECM-related genes in trisomic cells and also accounts for the decreased migration of trisomic fibroblasts [148]. Although the expression of RUNX1 in the adult heart is reported to be low, several studies have demonstrated that RUNX1 expression is increased in the context of cardiac pathologies [120,121,123,126], suggesting a possible role of RUNX1 in cardiac remodeling after heart failure [126].

## Figures and Tables

**Table 1:** ECM-related genes dysregulated in DS, possibly involved in the occurrence of CHD.

Gene Name	Location	Gene Product	Potential Role in CHD
*ADAMTS1* (ADAM Metallopeptidase with Thrombospondin Type 1 Motif 1)	21q21.3	Cleaves aggrecan, a cartilage proteoglycan, at the ‘1938-Glu-|-Leu-1939’ site (within the chondroitin sulfate attachment domain) and may be involved in its turnover. Has angiogenic inhibitor activity.	[23,105,106,107,108,109]
*ADAMTS5* (ADAM Metallopeptidase with Thrombospondin Type 1 Motif 5)	21q21.3	ECM-degrading enzyme that shows proteolytic activity towards the hyalectan group of chondroitin sulfate proteoglycans, including ACAN, VCAN, BCAN and NCAN. Plays an important role in connective tissue organization, development, inflammation and cell migration.	[23,109,110]
*COL6A1* (Collagen Type VI Alpha 1 Chain)/COL6A2 (Collagen Type VI Alpha 2 Chain)	21q22.3/21q22.3	Collagen VI acts as a cell-binding protein. Collagen VI is a ubiquitous nonfibrillar collagen composed of three chains (α1(VI), α2(VI), and α3(VI)) organized into a network of microfibrils important in anchoring the basement membrane to the ECM. Each chain contains a comparatively short triple helical domain with repeating Gly-X-Y subunits flanked by large globular von Willebrand factor type A domains.	[23,28,29,33,109,111,112,113,114]
*COL18A1* (Collagen Type XVIII Alpha 1 Chain)	21q22.3	Regulates ECM-dependent motility and morphogenesis of endothelial and non-endothelial cells. Inhibits angiogenesis by binding to the heparan sulfate proteoglycans involved in growth factor signaling. Inhibits VEGFA-induced endothelial cell proliferation and migration. Modulates endothelial cell migration in an integrin-dependent manner.	[23,109,115,116]
*DSCAM* (DS Cell Adhesion Molecule)	21q22.2	Plays a role in neuronal self-avoidance, promotes lamina-specific synaptic connections in the retina and mediates homophilic intercellular adhesion.	[14,27,33]
*JAM2* (Junctional Adhesion Molecule 2)	21q21.3	Mediates heterotypic cell–cell interactions with its cognate receptor JAM3 to regulate different cellular processes.Plays a role in homing and mobilization of hematopoietic stem and progenitor cells within the bone marrow. Plays a central role in leukocytes extravasation by facilitating not only transmigration, but also tethering and rolling of leukocytes along the endothelium. During myogenesis, it is involved in myocyte fusion.	[23,117,118]
*RUNX1* (Runt-Related Transcription Factor 1)	21q22.12	Required for the development of normal hematopoiesis. Forms the heterodimeric complex core-binding factor with CBFB. *RUNX* members modulate the transcription of their target genes. The heterodimers bind to the core site of several enhancers and promoters. Several studies have ascribed to RUNX1 an important role in regulating ECM genes, cell adhesion and migration.	[119,120,121,122,123,124,125,126]
*COL1A1* (Collagen Type I Alpha 1 Chain)/*COL1A2* (Collagen Type I Alpha 2 Chain)	17q21.33/7q21.3	Type I collagen is a member of group I collagen (fibrillar forming collagen). COL1A1 and COL1A2 genes provide instructions for making part of type I collagen. Type I collagen is the most abundant form of collagen in the human body.	[23,109,114,127]
*COL3A1* (Collagen Type III Alpha 1 Chain)	2q32.2	Involved in regulation of cortical development, it is the major ligand of ADGRG1 in the developing brain. Binding to ADGRG1 inhibits neuronal migration and activates the RhoA pathway by coupling ADGRG1 to GNA13 and possibly GNA12.	[23,109,128,129]
*COL5A1* (Collagen Type V Alpha 2 Chain)/*COL5A2* (Collagen Type V Alpha 2 Chain)	9q34.3/2q32.2	Type V collagen is a member of group I collagen (fibrillar-forming collagen). It is a minor connective tissue component of nearly ubiquitous distribution. Type V collagen binds to DNA, heparan sulfate, thrombospondin, heparin and insulin. Type V collagen is a key determinant in the assembly of tissue-specific matrices.	[23,109,114,130]
*COL15A1* (Collagen Type XV Alpha 1 Chain)	9q22.33	Structural protein that stabilizes microvessels and muscle cells, both in heart and in skeletal muscle.	[23,109,131,132,133]
*DDR1* (Discoidin Domain Receptor Tyrosine Kinase 1)	6p21.33	Functions as cell surface receptor for fibrillar collagen and regulates cell attachment to the ECM, remodeling of the ECM, cell migration, differentiation, survival and cell proliferation. Regulates remodeling of the ECM by upregulation of the metalloproteinases MMP2, MMP7 and MMP9, and thereby facilitates cell migration and wound healing.	[21]
*DDR2* (Discoidin Domain Receptor Tyrosine Kinase 2)	1q23.3	Required for normal bone development. Functions as cell surface receptor for fibrillar collagen and regulates cell differentiation, remodeling of the ECM, cell migration and cell proliferation. Regulates remodeling of the ECM by upregulation of the collagenases MMP1, MMP2 and MMP13, and thereby facilitates cell migration and tumor cell invasion. Promotes fibroblast migration and proliferation, and thereby contributes to cutaneous wound healing.	[23,32]
*FBLN1* (Fibulin 1)	22q13.31	Incorporated into fibronectin-containing matrix fibers. Plays a role in cell adhesion and migration along protein fibers within the ECM and contributes to the supramolecular organization of ECM architecture. Has been implicated in a role in cellular transformation and tumor invasion. Plays a role in hemostasis and thrombosis owing to its ability to bind fibrinogen and incorporate into clots.	[105,106,109,134,135]
*FN1* (Fibronectin 1)	2q35	Fibronectins bind cell surfaces and various compounds including collagen, fibrin, heparin, DNA and actin. Fibronectins are involved in cell adhesion, cell motility, opsonization, wound healing and maintenance of cell shape. Participates in the regulation of type I collagen deposition by osteoblasts.	[109,136,137,138,139]
*VCAN* (Versican)	5q14.2–q14.3	Plays a role in intercellular signaling and in connecting cells with the ECM. Takes part in the regulation of cell motility, growth and differentiation. Binds hyaluronic acid.	[106,107,109,140]

## Data Availability

Not applicable.

## References

[1] Van Der Linde D., Konings E.E.M., Slager M.A., Witsenburg M., Helbing W.A., Takkenberg J.J.M., Roos-Hesselink J.W. (2011). Birth Prevalence of Congenital Heart Disease Worldwide: A Systematic Review and Meta-Analysis. J. Am. Coll. Cardiol..

[2] Fahed A.C., Gelb B.D., Seidman J.G., Seidman C.E. (2013). Genetics of Congenital Heart Disease: The Glass Half Empty. Circ. Res..

[3] Urbano R. (2012). Health Issues in Persons with Down Syndrome.

[4] Stoll C., Dott B., Alembik Y., Roth M.-P. (2015). Associated congenital anomalies among cases with Down syndrome. Eur. J. Med. Genet..

[5] Maslen C.L., Babcock D., Robinson S.W., Bean L.J.H., Dooley K.J., Willour V.L., Sherman S.L. (2006). *CRELD1* mutations contribute to the occurrence of cardiac atrioventricular septal defects in Down syndrome. Am. J. Med. Genet. Part A.

[6] Asim A., Agarwal S. (2021). Congenital heart defects among Down’s syndrome cases: An updated review from basic research to an emerging diagnostics technology and genetic counselling. J. Genet..

[7] Rehman Y., Wazir H.D., Akbar A., Khan A.M., Hussain I., Afridi A., Gul H., Sadia H. (2022). Congenital Heart Disease and Its Association in Children with Down Syndrome. Cureus.

[8] Rahmani Z., Blouin J.L., Creau-Goldberg N., Watkins P.C., Mattei J.F., Poissonnier M., Prieur M., Chettouh Z., Nicole A., Aurias A. (1989). Critical role of the D21S55 region on chromosome 21 in the pathogenesis of Down syndrome. Proc. Natl. Acad. Sci. USA.

[9] McCormick M.K., Schinzel A., Petersen M.B., Stetten G., Driscoll D.J., Cantu E.S., Tranebjaerg L., Mikkelsen M., Watkins P.C., Antonarakis S.E. (1989). Molecular genetic approach to the characterization of the “Down syndrome region” of chromosome 21. Genomics.

[10] Korenberg J.R., Kawashima H., Pulst S.M., Ikeuchi T., Ogasawara N., Yamamoto K., Schonberg S.A., West R., Allen L., Magenis E. (1990). Molecular definition of a region of chromosome 21 that causes features of the Down syndrome phenotype. Am. J. Hum. Genet..

[11] Korenberg J.R., Bradley C., Disteche C.M. (1992). Down syndrome: Molecular mapping of the congenital heart disease and duodenal stenosis. Am. J. Hum. Genet..

[12] Delabar J.-M., Theophile D., Rahmani Z., Chettouh Z., Blouin J.-L., Prieur M., Noel B., Sinet P.-M. (1993). Molecular Mapping of Twenty-Four Features of Down Syndrome on Chromosome 21. Eur. J. Hum. Genet..

[13] Korenberg J.R., Chen X.N., Schipper R., Sun Z., Gonsky R., Gerwehr S., Carpenter N., Daumer C., Dignan P., Disteche C. (1994). Down syndrome phenotypes: The consequences of chromosomal imbalance. Proc. Natl. Acad. Sci. USA.

[14] Barlow G.M., Chen X.-N., Shi Z.Y., Lyons G.E., Kurnit D.M., Celle L., Spinner N.B., Zackai E., Pettenati M.J., Van Riper A.J. (2001). Down syndrome congenital heart disease: A narrowed region and a candidate gene. Anesth. Analg..

[15] Korbel J.O., Tirosh-Wagner T., Urban A.E., Chen X.N., Kasowski M., Dai L., Grubert F., Erdman C., Gao M.C., Lange K. (2009). The genetic architecture of Down syndrome phenotypes revealed by high-resolution analysis of human segmental trisomies. Proc. Natl. Acad Sci. USA.

[16] Pelleri M.C., Gennari E., Locatelli C., Piovesan A., Caracausi M., Antonaros F., Rocca A., Donati C.M., Conti L., Strippoli P. (2017). Genotype-phenotype correlation for congenital heart disease in Down syndrome through analysis of partial trisomy 21 cases. Genomics.

[17] Lyle R., Béna F., Gagos S., Gehrig C., Lopez G., Schinzel A., Lespinasse J., Bottani A., Dahoun S., Taine L. (2009). Genotype–phenotype correlations in Down syndrome identified by array CGH in 30 cases of partial trisomy and partial monosomy chromosome 21. Eur. J. Hum. Genet..

[18] Yu T., Li Z., Jia Z., Clapcote S.J., Liu C., Li S., Asrar S., Pao A., Chen R., Fan N. (2010). A mouse model of Down syndrome trisomic for all human chromosome 21 syntenic regions. Hum. Mol. Genet..

[19] Liu C., Belichenko P.V., Zhang L., Fu D., Kleschevnikov A.M., Baldini A., Antonarakis S.E., Mobley W.C., Yu Y.E. (2011). Mouse Models for Down Syndrome-Associated Developmental Cognitive Disabilities. Dev. Neurosci..

[20] Liu C., Morishima M., Jiang X., Yu T., Meng K., Ray D., Pao A., Ye P., Parmacek M.S., Yu Y.E. (2014). Engineered chromosome-based genetic mapping establishes a 3.7 Mb critical genomic region for Down syndrome-associated heart defects in mice. Hum. Genet..

[21] Lana-Elola E., Watson-Scales S., Slender A., Gibbins D., Martineau A., Douglas C., Mohun T., MC Fisher E., Tybulewicz V.L. (2016). Genetic dissection of Down syndrome-associated congenital heart defects using a new mouse mapping panel. eLife.

[22] Venegas-Zamora L., Bravo-Acuña F., Sigcho F., Gomez W., Bustamante-Salazar J., Pedrozo Z., Parra V. (2022). New Molecular and Organelle Alterations Linked to Down Syndrome Heart Disease. Front. Genet..

[23] Conti A., Fabbrini F., D’Agostino P., Negri R., Greco D., Genesio R., D’Armiento M., Olla C., Paladini D., Zannini M. (2007). Altered expression of mitochondrial and extracellular matrix genes in the heart of human fetuses with chromosome 21 trisomy. BMC Genom..

[24] Wright T.C., Orkin R.W., Destrempes M., Kurnit D.M. (1984). Increased adhesiveness of Down syndrome fetal fibroblasts in vitro. Proc. Natl. Acad. Sci. USA.

[25] Kurnit D.M., Aldridge J.F., Matsuoka R., Matthysse S., Opitz J.M., Reynolds J.F. (1985). Increased adhesiveness of trisomy 21 cells and atrioventricular canal malformations in down syndrome: A stochastic model. Am. J. Med. Genet..

[26] Agarwala K.L., Ganesh S., Amano K., Suzuki T., Yamakawa K. (2001). DSCAM, a Highly Conserved Gene in Mammals, Expressed in Differentiating Mouse Brain. Biochem. Biophys. Res. Commun..

[27] Dunlevy L., Bennett M., Slender A., Lana-Elola E., Tybulewicz V.L., Fisher E.M., Mohun T. (2010). Down’s syndrome-like cardiac developmental defects in embryos of the transchromosomic Tc1 mouse. Cardiovasc. Res..

[28] Davies G.E., Howard C.M., Farrer M.J., Coleman M.M., Bennett L.B., Cullen L.M., Wyse R.K.H., Burn J., Williamson R., Kessling A.M. (1995). Genetic variation in the *COL6A1* region is associated with congenital heart defects in trisomy 21 (Down’s syndrome). Ann. Hum. Genet..

[29] Groot A.C.G.-D., Bartram U., Oosthoek P.W., Bartelings M.M., Hogers B., Poelmann R.E., Jongewaard I.N., Klewer S.E. (2003). Collagen type VI expression during cardiac development and in human fetuses with trisomy 21. Anat. Rec..

[30] Vogel W., Gish G.D., Alves F., Pawson T. (1997). The Discoidin Domain Receptor Tyrosine Kinases Are Activated by Collagen. Mol. Cell.

[31] Faraci E., Eck M., Gerstmayer B., Bosio A., Vogel W.F. (2003). An extracellular matrix-specific microarray allowed the identification of target genes downstream of discoidin domain receptors. Matrix Biol..

[32] Morales M.O., Price R.L., Goldsmith E.C. (2005). Expression of Discoidin Domain Receptor 2 (DDR2) in the Developing Heart. Microsc. Microanal..

[33] Grossman T.R., Gamliel A., Wessells R.J., Taghli-Lamallem O., Jepsen K., Ocorr K., Korenberg J.R., Peterson K.L., Rosenfeld M.G., Bodmer R. (2011). Over-Expression of DSCAM and COL6A2 Cooperatively Generates Congenital Heart Defects. PLoS Genet..

[34] Kosaki R., Kosaki K., Matsushima K., Mitsui N., Matsumoto N., Ohashi H. (2005). Refining chromosomal region critical for Down syndrome-related heart defects with a case of cryptic 21q22.2 duplication. Congenit. Anomalies.

[35] Arron J.R., Winslow M.M., Polleri A., Chang C.-P., Wu H., Gao X., Neilson J.R., Chen L., Heit J.J., Kim S.K. (2006). NFAT dysregulation by increased dosage of *DSCR1* and *DYRK1A* on chromosome 21. Nature.

[36] Graef I.A., Chen F., Chen L., Kuo A., Crabtree G.R. (2001). Signals Transduced by Ca2+/Calcineurin and NFATc3/c4 Pattern the Developing Vasculature. Cell.

[37] Fuentes J.J., Genescà L., Kingsbury T.J., Cunningham K.W., Pérez-Riba M., Estivill X., de la Luna S. (2000). DSCR1, overexpressed in Down syndrome, is an inhibitor of calcineurin-mediated signaling pathways. Hum. Mol. Genet..

[38] Klee C.B., Ren H., Wang X. (1998). Regulation of the Calmodulin-stimulated Protein Phosphatase, Calcineurin. J. Biol. Chem..

[39] Flanagan W.M., Corthésy B., Bram R.J., Crabtree G.R. (1991). Nuclear association of a T-cell transcription factor blocked by FK-506 and cyclosporin A. Nature.

[40] Crabtree G.R., Olson E.N. (2002). NFAT Signaling: Choreographing the Social Lives of Cells. Cell.

[41] De La Pompa J.L., Timmerman L.A., Takimoto H., Yoshida H., Elia A.J., Samper E., Potter J., Wakeham A., Marengere L., Langille B.L. (1998). Role of the NF-ATc transcription factor in morphogenesis of cardiac valves and septum. Nature.

[42] Ranger A.M., Grusby M.J., Hodge M.R., Gravallese E.M., De La Brousse F.C., Hoey T., Mickanin C., Baldwin H.S., Glimcher L.H. (1998). The transcription factor NF-ATc is essential for cardiac valve formation. Nature.

[43] Chang C.-P., Neilson J.R., Bayle J.H., Gestwicki J.E., Kuo A., Stankunas K., Graef I.A., Crabtree G.R. (2004). A Field of Myocardial-Endocardial NFAT Signaling Underlies Heart Valve Morphogenesis. Cell.

[44] Casas C., Martínez S., Pritchard M., Fuentes J., Nadal M., Guimerà J., Arbonés M., Flórez J., Soriano E., Estivill X. (2001). Dscr1, a novel endogenous inhibitor of calcineurin signaling, is expressed in the primitive ventricle of the heart and during neurogenesis. Mech. Dev..

[45] Lange A.W., Molkentin J., E Yutzey K. (2004). *DSCR1* gene expression is dependent on NFATc1 during cardiac valve formation and colocalizes with anomalous organ development in trisomy 16 mice. Dev. Biol..

[46] Adayev T., Chen-Hwang M.-C., Murakami N., Wang R., Hwang Y.-W. (2006). MNB/DYRK1A phosphorylation regulates the interactions of synaptojanin 1 with endocytic accessory proteins. Biochem. Biophys. Res. Commun..

[47] Cousin M., Robinson P.J. (2001). The dephosphins: Dephosphorylation by calcineurin triggers synaptic vesicle endocytosis. Trends Neurosci..

[48] Fasano D., Parisi S., Pierantoni G.M., De Rosa A., Picillo M., Amodio G., Pellecchia M.T., Barone P., Moltedo O., Bonifati V. (2018). Alteration of endosomal trafficking is associated with early-onset parkinsonism caused by *SYNJ1* mutations. Cell Death Dis..

[49] Amodio G., Moltedo O., Fasano D., Zerillo L., Oliveti M., Di Pietro P., Faraonio R., Barone P., Pellecchia M.T., De Rosa A. (2019). PERK-Mediated Unfolded Protein Response Activation and Oxidative Stress in PARK20 Fibroblasts. Front. Neurosci..

[50] Choudhry H., Aggarwal M., Pan P.-Y. (2021). Mini-review: Synaptojanin 1 and its implications in membrane trafficking. Neurosci. Lett..

[51] Cossec J.-C., Lavaur J., Berman D.E., Rivals I., Hoischen A., Stora S., Ripoll C., Mircher C., Grattau Y., OlivoMarin J.-C. (2012). Trisomy for Synaptojanin1 in Down syndrome is functionally linked to the enlargement of early endosomes. Hum. Mol. Genet..

[52] De Rosa L., Fasano D., Zerillo L., Valente V., Izzo A., Mollo N., Amodio G., Polishchuk E., Polishchuk R., Melone M.A.B. (2022). Down Syndrome Fetal Fibroblasts Display Alterations of Endosomal Trafficking Possibly due to SYNJ1 Overexpression. Front. Genet..

[53] Curran J., Makara M.A., Mohler P.J. (2015). Endosome-based protein trafficking and Ca2+ homeostasis in the heart. Front. Physiol..

[54] Hardel N., Zolles G., Fakler B., Klöcker N. (2008). Recycling endosomes supply cardiac pacemaker channels for regulated surface expression. Cardiovasc. Res..

[55] Turan N.N., Moshal K.S., Roder K., Baggett B.C., Kabakov A.Y., Dhakal S., Teramoto R., Chiang D.Y.-E., Zhong M., Xie A. (2020). The endosomal trafficking regulator LITAF controls the cardiac Nav1.5 channel via the ubiquitin ligase NEDD4-2. J. Biol. Chem..

[56] Sailani M.R., Makrythanasis P., Valsesia A., Santoni F.A., Deutsch S., Popadin K., Borel C., Migliavacca E., Sharp A.J., Sail G.D. (2013). The complex SNP and CNV genetic architecture of the increased risk of congenital heart defects in Down syndrome. Genome Res..

[57] Calcagni G., Pugnaloni F., Digilio M., Unolt M., Putotto C., Niceta M., Baban A., Sparascio F.P., Drago F., De Luca A. (2021). Cardiac Defects and Genetic Syndromes: Old Uncertainties and New Insights. Genes.

[58] Balistreri C.R., Ammoscato C.L., Scola L., Fragapane T., Giarratana R.M., Lio D., Piccione M. (2020). Susceptibility to Heart Defects in Down Syndrome Is Associated with Single Nucleotide Polymorphisms in HAS 21 Interferon Receptor Cluster and *VEGFA* Genes. Genes.

[59] Robinson S.W., Morris C.D., Goldmuntz E., Reller M.D., Jones M.A., Steiner R.D., Maslen C.L. (2003). Missense Mutations in *CRELD1* Are Associated with Cardiac Atrioventricular Septal Defects. Am. J. Hum. Genet..

[60] Ackerman C., Locke A.E., Feingold E., Reshey B., Espana K., Thusberg J., Mooney S., Bean L.J., Dooley K.J., Cua C.L. (2012). An Excess of Deleterious Variants in VEGF-A Pathway Genes in Down-Syndrome-Associated Atrioventricular Septal Defects. Am. J. Hum. Genet..

[61] Asim A., Agarwal S., Panigrahi I., Sarangi A.N., Muthuswamy S., Kapoor A. (2018). *CRELD1* gene variants and atrioventricular septal defects in Down syndrome. Gene.

[62] Zhang H., Liu L., Tian J. (2019). Molecular mechanisms of congenital heart disease in down syndrome. Genes Dis..

[63] Reiter T.A., Abraham R.T., Choi M., Rusnak F. (1999). Redox regulation of calcineurin in T-lymphocytes. JBIC J. Biol. Inorg. Chem..

[64] Weston G., Haviv I., Rogers P. (2002). Microarray analysis of VEGF-responsive genes in myometrial endothelial cells. Mol. Hum. Reprod..

[65] Law E.W.L., Cheung A.K.L., Kashuba V.I., Pavlova T.V., Zabarovsky E.R., Lung H.L., Cheng Y., Chua D., Kwong D.L.-W., Tsao S.W. (2012). Anti-angiogenic and tumor-suppressive roles of candidate tumor-suppressor gene, *Fibulin-2*, in nasopharyngeal carcinoma. Oncogene.

[66] Person A.D., Garriock R.J., Krieg P.A., Runyan R.B., Klewer S.E. (2005). Frzb modulates Wnt-9a-mediated β-catenin signaling during avian atrioventricular cardiac cushion development. Dev. Biol..

[67] Armstrong E.J., Bischoff J. (2004). Heart Valve Development: Endothelial Cell Signaling and Differentiation. Circ. Res..

[68] Lambrechts D., Carmeliet P. (2004). Sculpting Heart Valves with NFATc and VEGF. Cell.

[69] Dor Y., Porat R., Keshet E., Godoy A., Montecinos V.P., Gray D.R., Sotomayor P., Yau J.M., Vethanayagam R.R., Singh S. (2001). Vascular endothelial growth factor and vascular adjustments to perturbations in oxygen homeostasis. Am. J. Physiol. Physiol..

[70] Kumar S.D., Yong S.-K., Dheen S.T., Bay B.-H., Tay S.S.-W. (2008). Cardiac Malformations Are Associated with Altered Expression of Vascular Endothelial Growth Factor and Endothelial Nitric Oxide Synthase Genes in Embryos of Diabetic Mice. Exp. Biol. Med..

[71] Hallaq H., Pinter E., Enciso J., McGrath J., Zeiss C., Brueckner M., Madri J., Jacobs H.C., Wilson C.M., Vasavada H. (2004). A null mutation of *Hhex* results in abnormal cardiac development, defective vasculogenesis and elevated Vegfa levels. Development.

[72] Montano M.M., Doughman Y.Q., Deng H., Chaplin L., Yang J., Wang N., Zhou Q., Ward N.L., Watanabe M. (2008). Mutation of the HEXIM1 Gene Results in Defects During Heart and Vascular Development Partly Through Downregulation of Vascular Endothelial Growth Factor. Circ. Res..

[73] Miquerol L., Langille B., Nagy A. (2000). Embryonic development is disrupted by modest increases in vascular endothelial growth factor gene expression. Development.

[74] Li H., Cherry S., Klinedinst D., DeLeon V., Redig J., Reshey B., Chin M.T., Sherman S.L., Maslen C.L., Reeves R.H. (2012). Genetic Modifiers Predisposing to Congenital Heart Disease in the Sensitized Down Syndrome Population. Circ. Cardiovasc. Genet..

[75] Alharbi K.M., Al-Mazroea A.H., Abdallah A.M., Almohammadi Y., Carlus S.J., Basit S. (2018). Targeted Next-Generation Sequencing of 406 Genes Identified Genetic Defects Underlying Congenital Heart Disease in Down Syndrome Patients. Pediatr. Cardiol..

[76] Piccoli C., Izzo A., Scrima R., Bonfiglio F., Manco R., Negri R., Quarato G., Cela O., Ripoli M., Prisco M. (2013). Chronic pro-oxidative state and mitochondrial dysfunctions are more pronounced in fibroblasts from Down syndrome foeti with congenital heart defects. Hum. Mol. Genet..

[77] Ong S.-B., Hausenloy D.J. (2010). Mitochondrial morphology and cardiovascular disease. Cardiovasc. Res..

[78] Liu Y., Huang Y., Xu C., An P., Luo Y., Jiao L., Luo J., Li Y. (2022). Mitochondrial Dysfunction and Therapeutic Perspectives in Cardiovascular Diseases. Int. J. Mol. Sci..

[79] Izzo A., Manco R., Bonfiglio F., Calì G., De Cristofaro T., Patergnani S., Cicatiello R., Scrima R., Zannini M., Pinton P. (2014). *NRIP1/RIP140* siRNA-mediated attenuation counteracts mitochondrial dysfunction in Down syndrome. Hum. Mol. Genet..

[80] Izzo A., Nitti M., Mollo N., Paladino S., Procaccini C., Faicchia D., Calì G., Genesio R., Bonfiglio F., Cicatiello R. (2017). Metformin restores the mitochondrial network and reverses mitochondrial dysfunction in Down syndrome cells. Hum. Mol. Genet..

[81] Mollo N., Nitti M., Zerillo L., Faicchia D., Micillo T., Accarino R., Secondo A., Petrozziello T., Calì G., Cicatiello R. (2019). Pioglitazone Improves Mitochondrial Organization and Bioenergetics in Down Syndrome Cells. Front. Genet..

[82] Mollo N., Cicatiello R., Aurilia M., Scognamiglio R., Genesio R., Charalambous M., Paladino S., Conti A., Nitsch L., Izzo A. (2020). Targeting Mitochondrial Network Architecture in Down Syndrome and Aging. Int. J. Mol. Sci..

[83] Izzo A., Mollo N., Nitti M., Paladino S., Calì G., Genesio R., Bonfiglio F., Cicatiello R., Barbato M., Sarnataro V. (2018). Mitochondrial dysfunction in down syndrome: Molecular mechanisms and therapeutic targets. Mol. Med..

[84] Stennard F.A., Costa M.W., Elliott D.A., Rankin S., Haast S.J.P., Lai D., McDonald L.P.A., Niederreither K., Dolle P., Bruneau B.G. (2003). Cardiac T-box factor Tbx20 directly interacts with Nkx2-5, GATA4, and GATA5 in regulation of gene expression in the developing heart. Dev. Biol..

[85] Cai X., Nomura-Kitabayashi A., Cai W., Yan J., Christoffels V.M., Cai C.-L. (2011). Myocardial Tbx20 regulates early atrioventricular canal formation and endocardial epithelial–mesenchymal transition via Bmp2. Dev. Biol..

[86] Zhao Y., Samal E., Srivastava D. (2005). Serum response factor regulates a muscle-specific microRNA that targets Hand2 during cardiogenesis. Nature.

[87] Deshpande A., Shetty P.M.V., Frey N., Rangrez A.Y. (2022). SRF: A seriously responsible factor in cardiac development and disease. J. Biomed. Sci..

[88] Coppola A., Romito A., Borel C., Gehrig C., Gagnebin M., Falconnet E., Izzo A., Altucci L., Banfi S., Antonarakis S. (2014). Cardiomyogenesis is controlled by the miR-99a/let-7c cluster and epigenetic modifications. Stem Cell Res..

[89] Brás A., Rodrigues A.S., Gomes B., Rueff J. (2018). Down syndrome and microRNAs (Review). Biomed. Rep..

[90] Latronico M.V.G., Catalucci D., Condorelli G. (2008). MicroRNA and Cardiac Pathologies. Physiol. Genom..

[91] Izzo A., Manco R., de Cristofaro T., Bonfiglio F., Cicatiello R., Mollo N., De Martino M., Genesio R., Zannini M., Conti A. (2017). Overexpression of Chromosome 21 miRNAs May Affect Mitochondrial Function in the Hearts of Down Syndrome Fetuses. Int. J. Genom..

[92] Mollo N., Izzo A., Nitti M., Paladino S., Calì G., Genesio R., Bonfiglio F., Cicatiello R., Conti A., Nitsch L. (2017). Mitochondria in Down Syndrome: From Organelle Abnormalities to Clinical Phenotype. Down Syndrome.

[93] Quiñones-Lombraña A., Blanco J.G. (2015). Chromosome 21-derived hsa-miR-155-5p regulates mitochondrial biogenesis by targeting Mitochondrial Transcription Factor A (TFAM). Biochim. et Biophys. Acta (BBA)-Mol. Basis Dis..

[94] Ono K., Kuwabara Y., Han J. (2011). MicroRNAs and cardiovascular diseases. FEBS J..

[95] Sayed D., Hong C., Chen I.-Y., Lypowy J., Abdellatif M. (2007). MicroRNAs Play an Essential Role in the Development of Cardiac Hypertrophy. Circ. Res..

[96] Ramachandran D., Zeng Z., Locke A.E., Mulle J.G., Bean L.J., Rosser T.C., Dooley K.J., Cua C.L., Capone G.T., Reeves R.H. (2015). Genome-Wide Association Study of Down Syndrome-Associated Atrioventricular Septal Defects. G3 Genes Genomes Genet..

[97] Just S., Hirth S., Berger I.M., Fishman M.C., Rottbauer W. (2016). The mediator complex subunit Med10 regulates heart valve formation in zebrafish by controlling Tbx2b-mediated Has2 expression and cardiac jelly formation. Biochem. Biophys. Res. Commun..

[98] Koch C.D., Lee C.M., Apte S.S. (2020). Aggrecan in Cardiovascular Development and Disease. J. Histochem. Cytochem..

[99] Bloksgaard M., Lindsey M., Martinez-Lemus L.A. (2018). Extracellular matrix in cardiovascular pathophysiology. Am. J. Physiol. Heart Circ. Physiol..

[100] Stevens M.V., Parker P., Vaillancourt R.R., Camenisch T.D. (2006). MEKK4 regulates developmental EMT in the embryonic heart. Dev. Dyn..

[101] Shelton E.L., Yutzey K.E. (2007). Tbx20 regulation of endocardial cushion cell proliferation and extracellular matrix gene expression. Dev. Biol..

[102] Vilardell M., Rasche A., Thormann A., Maschke-Dutz E., Pérez-Jurado L.A., Lehrach H., Herwig R. (2011). Meta-analysis of heterogeneous Down Syndrome data reveals consistent genome-wide dosage effects related to neurological processes. BMC Genom..

[103] Vilardell M., Civit S., Herwig R. (2013). An integrative computational analysis provides evidence for FBN1-associated network deregulation in trisomy 21. Biol. Open.

[104] De Toma I., Sierra C., Dierssen M. (2021). Meta-analysis of transcriptomic data reveals clusters of consistently deregulated gene and disease ontologies in Down syndrome. PLoS Comput. Biol..

[105] Twal W.O., Czirok A., Hegedus B., Knaak C., Chintalapudi M.R., Okagawa H., Sugi Y., Argraves W.S. (2001). Fibulin-1 suppression of fibronectin-regulated cell adhesion and motility. J. Cell Sci..

[106] Kern C.B., Twal W.O., Mjaatvedt C.H., Fairey S.E., Toole B.P., Iruela-Arispe M.L., Argraves W.S. (2006). Proteolytic cleavage of versican during cardiac cushion morphogenesis. Dev. Dyn..

[107] Kern C.B., Norris R.A., Thompson R.P., Argraves W.S., Fairey S.E., Reyes L., Hoffman S., Markwald R.R., Mjaatvedt C.H. (2007). Versican proteolysis mediates myocardial regression during outflow tract development. Dev. Dyn..

[108] Stankunas K., Hang C.T., Tsun Z.-Y., Chen H., Lee N.V., Wu J.I., Shang C., Bayle J.H., Shou W., Iruela-Arispe M.L. (2008). Endocardial Brg1 Represses *ADAMTS1* to Maintain the Microenvironment for Myocardial Morphogenesis. Dev. Cell.

[109] Lockhart M., Wirrig E., Phelps A., Wessels A. (2011). Extracellular matrix and heart development. Birth Defects Res. Part A Clin. Mol. Teratol..

[110] McCulloch D.R., Le Goff C., Bhatt S., Dixon L.J., Sandy J.D., Apte S.S. (2009). *Adamts5*, the gene encoding a proteoglycan-degrading metalloprotease, is expressed by specific cell lineages during mouse embryonic development and in adult tissues. Gene Expr. Patterns.

[111] Fuentes J., Pritchard M., Estivill X. (1997). Genomic Organization, Alternative Splicing, and Expression Patterns of the *DSCR1* (Down Syndrome Candidate Region 1) Gene. Genomics.

[112] Bonaldo P., Braghetta P., Zanetti M., Piccolo S., Volpin D., Bressan G.M. (1998). Collagen VI deficiency induces early onset myopathy in the mouse: An animal model for Bethlem myopathy. Hum. Mol. Genet..

[113] Klewer S.E., Krob S.L., Kolker S.J., Kitten G.T. (1998). Expression of Type VI Collagen in the Developing Mouse Heart. Dev. Dyn..

[114] Kruithof B.P., Krawitz S.A., Gaussin V. (2007). Atrioventricular valve development during late embryonic and postnatal stages involves condensation and extracellular matrix remodeling. Dev. Biol..

[115] Utriainen A., Sormunen R., Kettunen M., Carvalhaes L.S., Sajanti E., Eklund L., Kauppinen R., Kitten G.T., Pihlajaniemi T. (2004). Structurally altered basement membranes and hydrocephalus in a type XVIII collagen deficient mouse line. Hum. Mol. Genet..

[116] Carvalhaes L.S., Gervásio O.L., Guatimosim C., Heljasvaara R., Sormunen R., Pihlajaniemi T., Kitten G.T. (2006). Collagen XVIII/endostatin is associated with the epithelial-mesenchymal transformation in the atrioventricular valves during cardiac development. Dev. Dyn..

[117] Reynolds L.E., Watson A.R., Baker M., Jones T.A., D’Amico G., Robinson S.D., Joffre C., Garrido-Urbani S., Rodriguez-Manzaneque J.C., Martino-Echarri E. (2010). Tumour angiogenesis is reduced in the Tc1 mouse model of Down’s syndrome. Nature.

[118] Li H., Edie S., Klinedinst D., Jeong J.S., Blackshaw S., Maslen C.L., Reeves R.H. (2016). Penetrance of Congenital Heart Disease in a Mouse Model of Down Syndrome Depends on a Trisomic Potentiator of a Disomic Modifier. Genetics.

[119] Levanon D., Brenner O., Negreanu V., Bettoun D., Woolf E., Eilam R., Lotem J., Gat U., Otto F., Speck N. (2001). Spatial and temporal expression pattern of Runx3 (Aml2) and Runx1 (Aml1) indicates non-redundant functions during mouse embryogenesis. Mech. Dev..

[120] Gattenlöhner S., Waller C., Ertl G., Bültmann B.-D., Müller-Hermelink H.-K., Marx A. (2003). NCAM(CD56) and RUNX1(AML1) Are Up-Regulated in Human Ischemic Cardiomyopathy and a Rat Model of Chronic Cardiac Ischemia. Am. J. Pathol..

[121] Kubin T., Pöling J., Kostin S., Gajawada P., Hein S., Rees W., Wietelmann A., Tanaka M., Lörchner H., Schimanski S. (2011). Oncostatin M Is a Major Mediator of Cardiomyocyte Dedifferentiation and Remodeling. Cell Stem Cell.

[122] Eulalio A., Mano M., Ferro M.D., Zentilin L., Sinagra G., Zacchigna S., Giacca M. (2012). Functional screening identifies miRNAs inducing cardiac regeneration. Nature.

[123] Wang Y., Ma S., Wang Q., Hu W., Wang D., Li X., Su T., Qin X., Zhang X., Ma K. (2014). Effects of cannabinoid receptor type 2 on endogenous myocardial regeneration by activating cardiac progenitor cells in mouse infarcted heart. Sci. China Life Sci..

[124] Lluri G., Huang V., Touma M., Liu X., Harmon A.W., Nakano A. (2015). Hematopoietic progenitors are required for proper development of coronary vasculature. J. Mol. Cell. Cardiol..

[125] Górnikiewicz B., Ronowicz A., Krzemiński M., Sachadyn P. (2016). Changes in gene methylation patterns in neonatal murine hearts: Implications for the regenerative potential. BMC Genom..

[126] McCarroll C.S., He W., Foote K., Bradley A., Mcglynn K., Vidler F., Nixon C., Nather K., Fattah C., Riddell A. (2018). Runx1 Deficiency Protects Against Adverse Cardiac Remodeling After Myocardial Infarction. Circulation.

[127] Lincoln J., Alfieri C.M., Yutzey K.E. (2004). Development of heart valve leaflets and supporting apparatus in chicken and mouse embryos. Dev. Dyn..

[128] Liu X., Wu H., Byrne M., Krane S., Jaenisch R. (1997). Type III collagen is crucial for collagen I fibrillogenesis and for normal cardiovascular development. Proc. Natl. Acad. Sci. USA.

[129] Tan M., Wang X., Liu H., Peng X., Yang Y., Yu H., Xu L., Li J., Cao H. (2022). Genetic Diagnostic Yield and Novel Causal Genes of Congenital Heart Disease. Front. Genet..

[130] Wenstrup R.J., Florer J.B., Brunskill E.W., Bell S.M., Chervoneva I., Birk D.E. (2004). Type V Collagen Controls the Initiation of Collagen Fibril Assembly. J. Biol. Chem..

[131] Eklund L., Piuhola J., Komulainen J., Sormunen R., Ongvarrasopone C., Fässler R., Muona A., Ilves M., Ruskoaho H., Takala T.E.S. (2001). Lack of type XV collagen causes a skeletal myopathy and cardiovascular defects in mice. Proc. Natl. Acad. Sci. USA.

[132] Muona A., Eklund L., Väisänen T., Pihlajaniemi T. (2002). Developmentally regulated expression of type XV collagen correlates with abnormalities in Col15a1−/− mice. Matrix Biol..

[133] Rasi K., Piuhola J., Czabanka M., Sormunen R., Ilves M., Leskinen H., Rysä J., Kerkelä R., Janmey P., Heljasvaara R. (2010). Collagen XV Is Necessary for Modeling of the Extracellular Matrix and Its Deficiency Predisposes to Cardiomyopathy. Circ. Res..

[134] Spence S., Argraves W., Walters L., Hungerford J.E., Little C.D. (1992). Fibulin is localized at sites of epithelial-mesenchymal transitions in the early avian embryo. Dev. Biol..

[135] Cooley M.A., Kern C.B., Fresco V.M., Wessels A., Thompson R.P., McQuinn T.C., Twal W.O., Mjaatvedt C.H., Drake C.J., Argraves W.S. (2008). Fibulin-1 is required for morphogenesis of neural crest-derived structures. Dev. Biol..

[136] Mjaatvedt C., Lepera R., Markwald R. (1987). Myocardial specificity for initiating endothelial-mesenchymal cell transition in embryonic chick heart correlates with a particulate distribution of fibronectin. Dev. Biol..

[137] Ffrench-Constant C., Hynes R. (1989). Alternative splicing of fibronectin is temporally and spatially regulated in the chicken embryo. Development.

[138] Icardo J.M., Nakamura A., Fernandez-Teran M.A., Manasek F.J. (1992). Effects of injecting fibronectin and antifibronectin antibodies on cushion mesenchyme formation in the chick. Anat. Embryol..

[139] Astrof S., Crowley D., Hynes R.O. (2007). Multiple cardiovascular defects caused by the absence of alternatively spliced segments of fibronectin. Dev. Biol..

[140] Chan C.K., Rolle M.W., Potter-Perigo S., Braun K.R., Van Biber B.P., Laflamme M.A., Murry C.E., Wight T.N. (2010). Differentiation of cardiomyocytes from human embryonic stem cells is accompanied by changes in the extracellular matrix production of versican and hyaluronan. J. Cell. Biochem..

[141] Delom F., Burt E., Hoischen A., Veltman J., Groet J., Cotter F.E., Nizetic D. (2009). Transchromosomic cell model of Down syndrome shows aberrant migration, adhesion and proteome response to extracellular matrix. Proteome Sci..

[142] Marino B. (1993). Congenital heart disease in patients with Down’s Syndrome: Anatomic and genetic aspects. Biomed. Pharmacother..

[143] Jongewaard I.N., Lauer R.M., Behrendt D.A., Patil S., Klewer S.E. (2002). Beta 1 integrin activation mediates adhesive differences between trisomy 21 and non-trisomic fibroblasts on type VI collagen. Am. J. Med. Genet..

[144] Aumailley M., Specks U., Timpl R. (1991). Cell Adhesion to Type-VI Collagen. Biochem. Soc. Trans..

[145] Pankov R., Yamada K.M. (2002). Fibronectin at a glance. J. Cell Sci..

[146] George E., Georges-Labouesse E., Patel-King R., Rayburn H., Hynes R. (1993). Defects in mesoderm, neural tube and vascular development in mouse embryos lacking fibronectin. Development.

[147] Lee N.V., Rodriguez-Manzaneque J.C., Thai S.N.-M., Twal W.O., Luque A., Lyons K.M., Argraves W.S., Iruela-Arispe M.L. (2005). Fibulin-1 Acts as a Cofactor for the Matrix Metalloprotease ADAMTS-1. J. Biol. Chem..

[148] Mollo N., Aurilia M., Scognamiglio R., Zerillo L., Cicatiello R., Bonfiglio F., Pagano P., Paladino S., Conti A., Nitsch L. (2022). Overexpression of the Hsa21 Transcription Factor RUNX1 Modulates the Extracellular Matrix in Trisomy 21 Cells. Front. Genet..

[149] De Cegli R., Romito A., Iacobacci S., Mao L., Lauria M., Fedele A.O., Klose J., Borel C., Descombes P., Antonarakis S.E. (2010). A mouse embryonic stem cell bank for inducible overexpression of human chromosome 21 genes. Genome Biol..

[150] Levanon D., Groner Y. (2004). Structure and regulated expression of mammalian *RUNX* genes. Oncogene.

[151] Lie-A-Ling M., Marinopoulou E., Li Y., Patel R., Stefanska M., Bonifer C., Miller C., Kouskoff V., Lacaud G. (2014). RUNX1 positively regulates a cell adhesion and migration program in murine hemogenic endothelium prior to blood emergence. Blood.

[152] Michaud J., Simpson K.M., Escher R., Buchet-Poyau K., Beissbarth T., Carmichael C., Ritchie M.E., Schütz F., Cannon P., Liu M. (2008). Integrative analysis of RUNX1 downstream pathways and target genes. BMC Genom..

[153] Wotton S., Terry A., Kilbey A., Jenkins A., Herzyk P., Cameron E., Neil J.C. (2008). Gene array analysis reveals a common Runx transcriptional programme controlling cell adhesion and survival. Oncogene.

[154] Barutcu A.R., Hong D., Lajoie B.R., McCord R.P., van Wijnen A.J., Lian J.B., Stein J.L., Dekker J., Imbalzano A.N., Stein G.S. (2016). RUNX1 contributes to higher-order chromatin organization and gene regulation in breast cancer cells. Biochim. et Biophys. Acta (BBA)-Gene Regul. Mech..

[155] Sobol M., Klar J., Laan L., Shahsavani M., Schuster J., Annerén G., Konzer A., Mi J., Bergquist J., Nordlund J. (2019). Transcriptome and Proteome Profiling of Neural Induced Pluripotent Stem Cells from Individuals with Down Syndrome Disclose Dynamic Dysregulations of Key Pathways and Cellular Functions. Mol. Neurobiol..

[156] Zheng Q., Zhou G., Morello R., Chen Y., Garcia-Rojas X., Lee B. (2003). Type X collagen gene regulation by Runx2 contributes directly to its hypertrophic chondrocyte–specific expression in vivo. J. Cell Biol..

[157] Higashikawa A., Saito T., Ikeda T., Kamekura S., Kawamura N., Kan A., Oshima Y., Ohba S., Ogata N., Takeshita K. (2009). Identification of the core element responsive to runt-related transcription factor 2 in the promoter of human type x collagen gene. Arthritis Rheum..

[158] Wei J., Shimazu J., Makinistoglu M.P., Maurizi A., Kajimura D., Zong H., Takarada T., Iezaki T., Pessin J.E., Hinoi E. (2015). Glucose Uptake and Runx2 Synergize to Orchestrate Osteoblast Differentiation and Bone Formation. Cell.

[159] Wang T., Jin H., Hu J., Li X., Ruan H., Xu H., Wei L., Dong W., Teng F., Gu J. (2020). COL4A1 promotes the growth and metastasis of hepatocellular carcinoma cells by activating FAK-Src signaling. J. Exp. Clin. Cancer Res..

[160] Sarohi V., Chakraborty S., Basak T. (2022). Exploring the cardiac ECM during fibrosis: A new era with next-gen proteomics. Front. Mol. Biosci..

[161] Li Q., Lai Q., He C., Fang Y., Yan Q., Zhang Y., Wang X., Gu C., Wang Y., Ye L. (2019). RUNX1 promotes tumour metastasis by activating the Wnt/β-catenin signalling pathway and EMT in colorectal cancer. J. Exp. Clin. Cancer Res..

[162] Keita M., Bachvarova M., Morin C., Plante M., Gregoire J., Renaud M.-C., Sebastianelli A., Trinh X.B., Bachvarov D. (2013). The RUNX1 transcription factor is expressed in serous epithelial ovarian carcinoma and contributes to cell proliferation, migration and invasion. Cell Cycle.

[163] Planagumà J., Liljeström M., Alameda F., Butzow R., Virtanen I., Reventós J., Hukkanen M. (2011). Matrix metalloproteinase-2 and matrix metalloproteinase-9 codistribute with transcription factors RUNX1/AML1 and ETV5/ERM at the invasive front of endometrial and ovarian carcinoma. Hum. Pathol..

[164] Sangpairoj K., Vivithanaporn P., Apisawetakan S., Chongthammakun S., Sobhon P., Chaithirayanon K. (2017). RUNX1 Regulates Migration, Invasion, and Angiogenesis via p38 MAPK Pathway in Human Glioblastoma. Cell. Mol. Neurobiol..

[165] Laufer B.I., Hwang H., Jianu J.M., Mordaunt C.E., Korf I.F., Hertz-Picciotto I., LaSalle J.M. (2020). Low-pass whole genome bisulfite sequencing of neonatal dried blood spots identifies a role for RUNX1 in Down syndrome DNA methylation profiles. Hum. Mol. Genet..

[166] Dobosz A., Grabowska A., Bik-Multanowski M. (2019). Hypermethylation of *NRG1* gene correlates with the presence of heart defects in Down’s syndrome. J. Genet..

[167] Mollo N., Esposito M., Aurilia M., Scognamiglio R., Accarino R., Bonfiglio F., Cicatiello R., Charalambous M., Procaccini C., Micillo T. (2021). Human Trisomic iPSCs from Down Syndrome Fibroblasts Manifest Mitochondrial Alterations Early during Neuronal Differentiation. Biology.

[168] Bonnans C., Chou J., Werb Z. (2014). Remodelling the extracellular matrix in development and disease. Nat. Rev. Mol. Cell Biol..

[169] Yamada K.M., Collins J.W., Walma D.A.C., Doyle A.D., Morales S.G., Lu J., Matsumoto K., Nazari S.S., Sekiguchi R., Shinsato Y. (2019). Extracellular matrix dynamics in cell migration, invasion and tissue morphogenesis. Int. J. Exp. Pathol..

[170] Garcia O., Torres M., Helguera P., Coskun P., Busciglio J. (2010). A Role for Thrombospondin-1 Deficits in Astrocyte-Mediated Spine and Synaptic Pathology in Down’s Syndrome. PLoS ONE.

[171] Danopoulos S., Bhattacharya S., Deutsch G., Nih L.R., Slaunwhite C., Mariani T.J., Al Alam D. (2021). Prenatal histological, cellular, and molecular anomalies in Trisomy 21 lung. J. Pathol..

[172] Von Kaisenberg C.S., Krenn V., Ludwig M., Nicolaides K., Brand-Saberi B. (1998). Morphological classification of nuchal skin in human fetuses with trisomy 21, 18, and 13 at 12–18 weeks and in a trisomy 16 mouse. Anat. Embryol..

[173] Wirrig E.E., Snarr B.S., Chintalapudi M.R., O’Neal J.L., Phelps A.L., Barth J.L., Fresco V.M., Kern C.B., Mjaatvedt C.H., Toole B.P. (2007). Cartilage link protein 1 (Crtl1), an extracellular matrix component playing an important role in heart development. Dev. Biol..

[174] Warde-Farley D., Donaldson S.L., Comes O., Zuberi K., Badrawi R., Chao P., Franz M., Grouios C., Kazi F., Lopes C.T. (2010). The GeneMANIA prediction server: Biological network integration for gene prioritization and predicting gene function. Nucleic Acids Res..

[175] Franz M., Rodriguez H., Lopes C., Zuberi K., Montojo J., Bader G.D., Morris Q. (2018). GeneMANIA update 2018. Nucleic Acids Res..

